# Supplementary material for: Two doses of SARS-CoV-2 vaccination induce robust immune responses to emerging SARS-CoV-2 variants of concern
Source: Nat Commun. 2021 Aug 17;12:5061. doi: 10.1038/s41467-021-25167-5 (PMC8371089; doi:10.1038/s41467-021-25167-5)
Supplement: Supplementary file 1 — Supplementary information [file 41467_2021_25167_MOESM1_ESM.pdf]

# Supplementary Figure 1

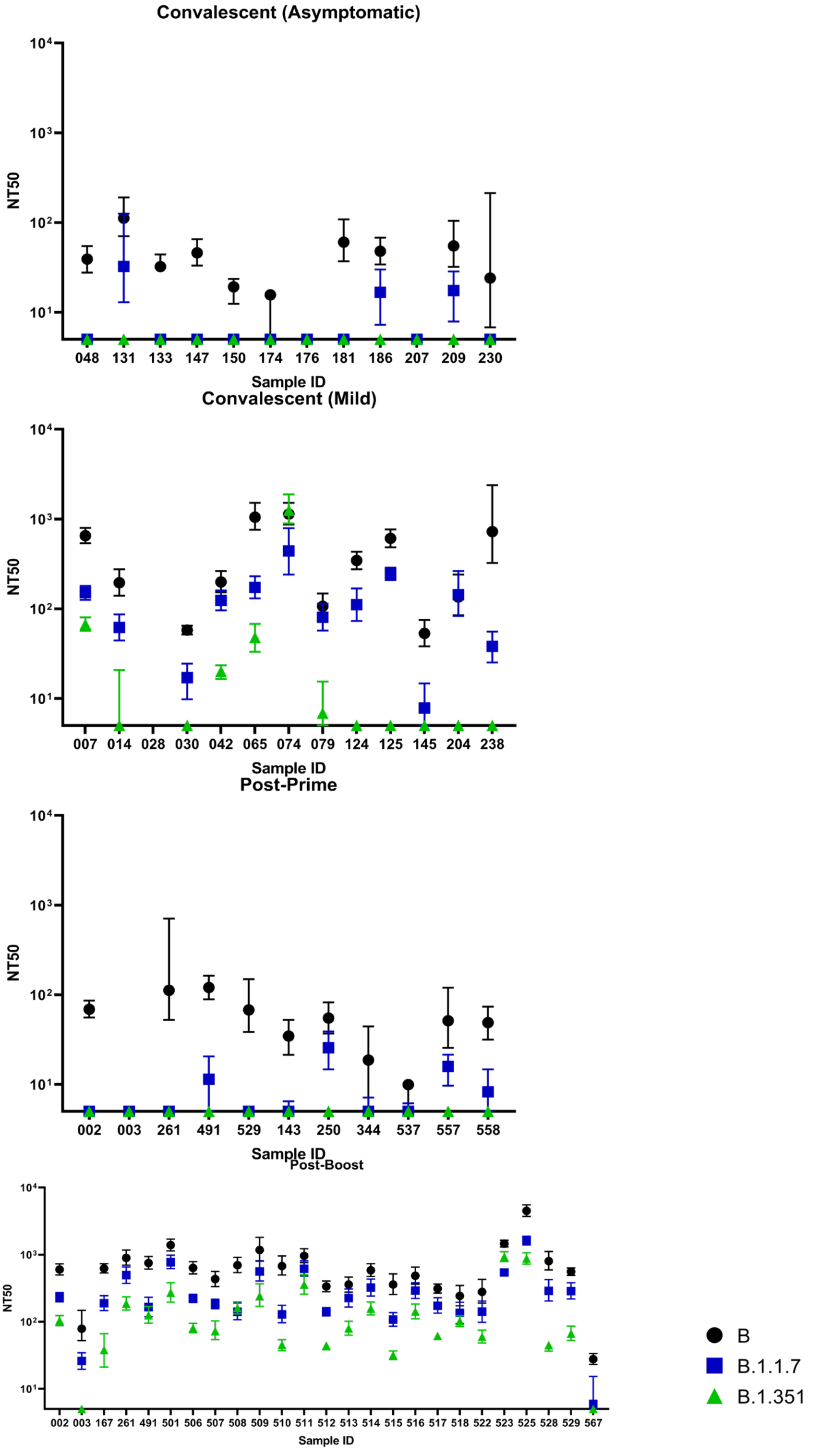


**Individual neutralization parameters.** For each donor in the study (indicated by their respective Sample ID) the neutralizing potency of their serum (NT50, ± 95% confidence intervals, derived by non-linear regression from the titration curves shown in S2) against isolates of lineage B, B.1.1.7 and B.1.351 is plotted. Where no neutralization could be reliably determined, the corresponding symbol is placed at the limit of detection, on the x-axis. Vaccinees post-prime n=11; vaccinees post-boost n=25; asymptomatic COVID-19 convalescents n=12; mild COVID-19 convalescents n=13.

# Supplementary Figure 2

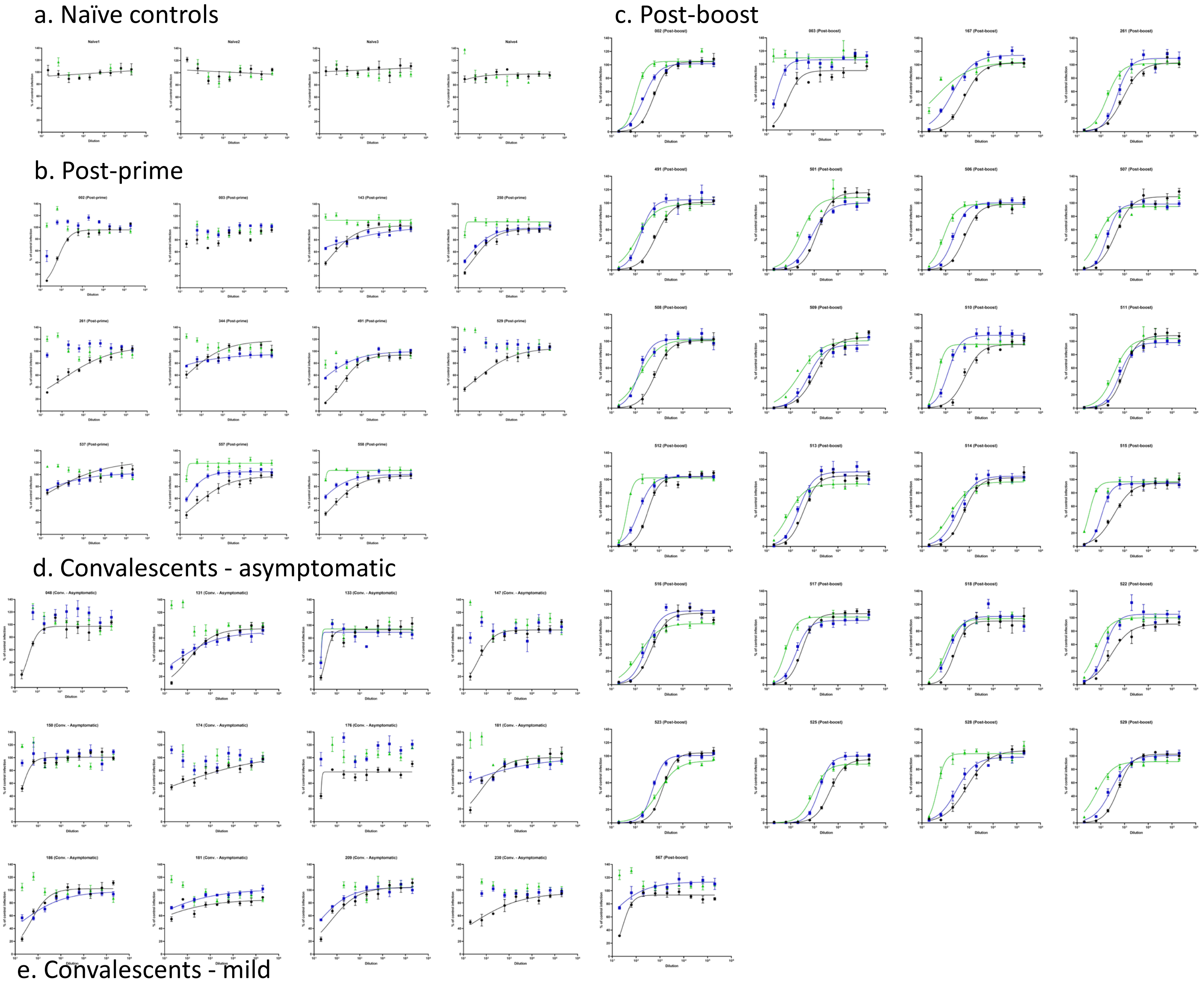


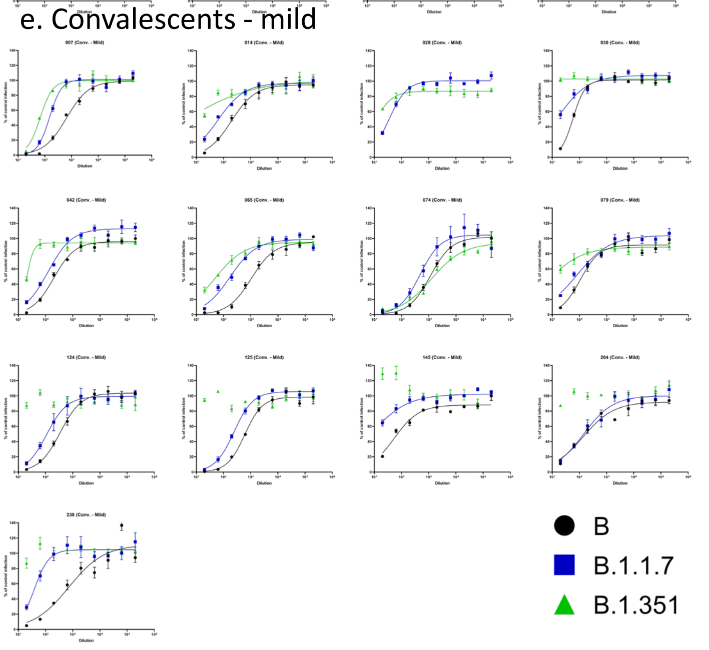


**Individual neutralization curves for each antibody/isolate combination**

The number of infectious foci generated by replicate samples of a standardized preparation of virus (isolates of lineages B, B.1.1.7 and B.1.351), following incubation with ten dilutions of each donor's serum ($log_{10}$0.5-fold dilution series) in Vero cells (ATCC CCL-81) was determined by immunostaining. For each serum/isolate combination, mean number of foci ± SD relative to a no-antibody control is plotted against the reciprocal of the respective serum dilution (i.e. $10^3$ corresponds to a 1/1000 dilution of serum). Data were fitted by non-linear regression in GraphPad Prism 9 to the Hill Equation, with TOP and BOTTOM constrained to 100% and 0%, respectively. Where a significant fit was obtained, it is represented by a trend line on the respective plot, and NT50 values used in Figure S1 and main results. **S2a** Sera from four naïve control donors. **S2b** Sera from eleven post-prime donors. **S2c** Sera from 25 post-boost donors. **S2d** Sera from twelve asymptomatic PCR positive convalescent donors. **S2e** Sera from thirteen convalescent donors with mild COVID-19 symptoms.

# Supplementary Figure 3

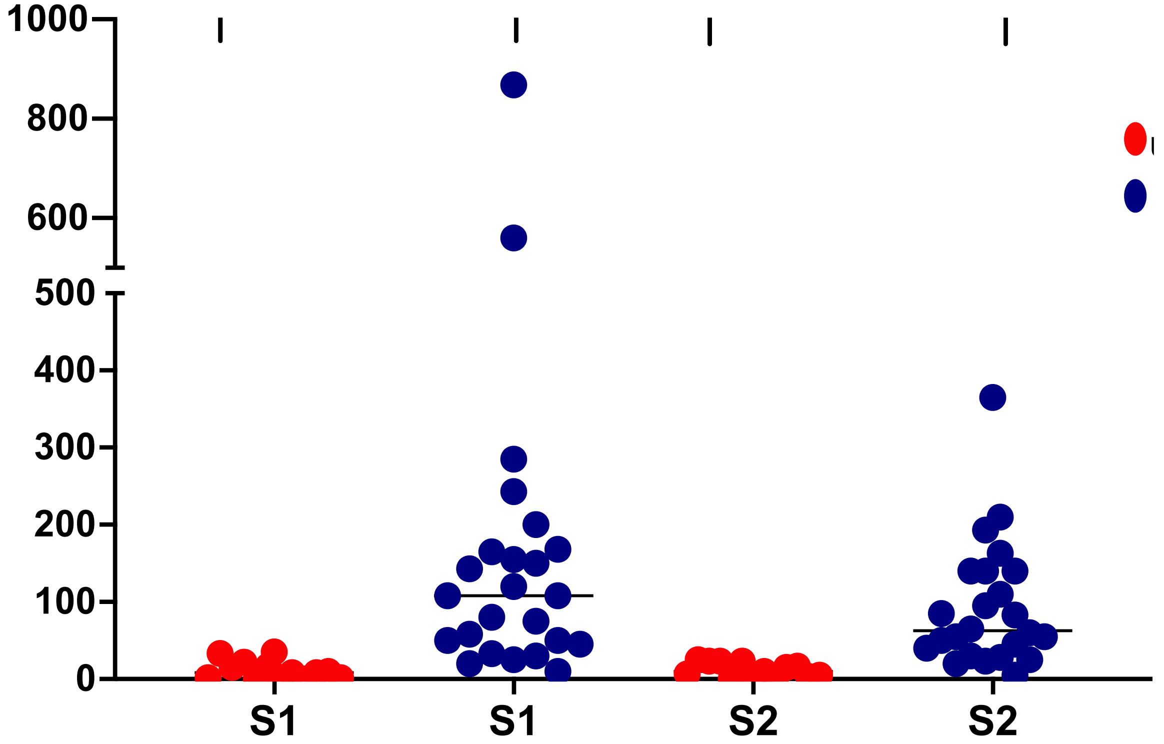


**S1 and S2 responses in unvaccinated volunteers and volunteers after two doses of BNT162b2 vaccine**

Ex vivo IFN-γ ELISpot T cell responses to S1 and S2 peptides in unvaccinated volunteers and volunteers after two doses of BNT162b2 vaccine. SFC/$10^6$ PBMC = spot forming cells per million peripheral blood mononuclear cells, with background subtracted. Samples are unpaired and comparison of two groups was performed using two-tailed Mann-Whitney U test. ns = not significant, * = <0.05, ** = <0.01, *** = < 0.001 and **** = <0.0001. n = 13 for unvaccinated participants, n = 24 for participants who received two vaccine doses. Lines indicate group medians.

# Supplementary Figure 4

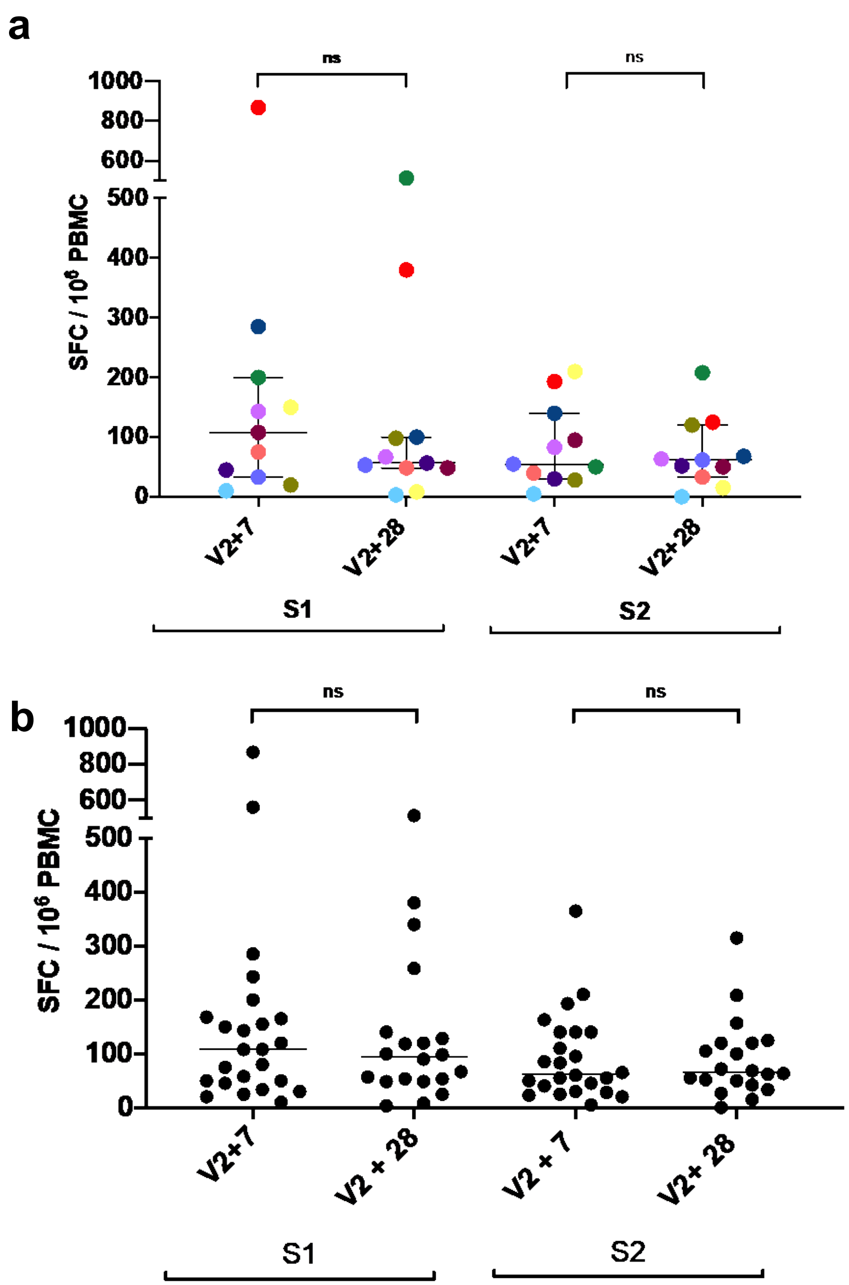


**S1 and S2 responses in volunteers after 7 days and 28 days after second dose of BNT162b2.**
Ex vivo IFN-γ ELISpot T cell responses were measured using IFN-γ ELISpot assays to S1 and S2 peptides in volunteers 7 days and 28 days after second dose of BNT162b2 vaccine **a** Paired T cell responses in 11 volunteers, each color represents the same individual **b** T cell responses in 24 volunteers. SFC/$10^6$ PBMC = spot forming cells per million peripheral blood mononuclear cells, with background subtracted. Comparison of two groups was performed using two-tailed Mann-Whitney U test. ns = not significant.

# Supplementary Figure 5

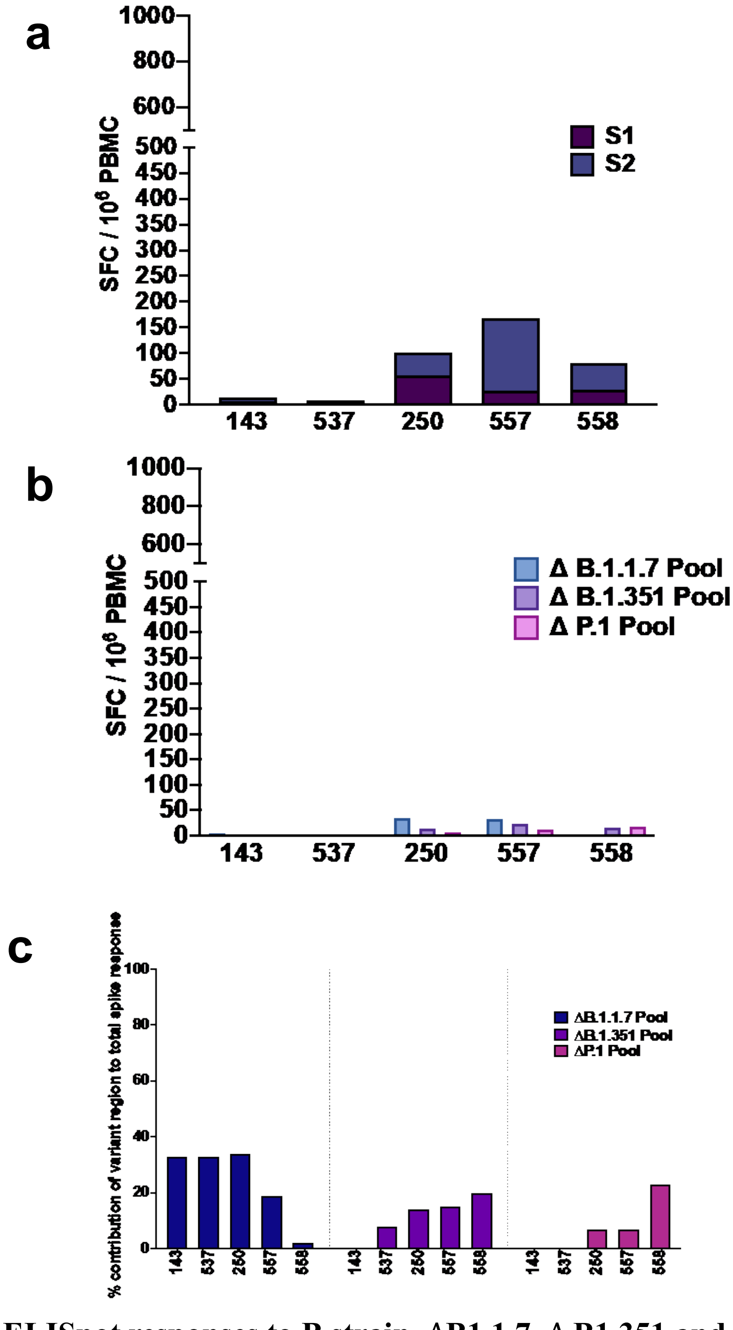


**IFN-γ ELISpot responses to B strain, ΔB1.1.7, Δ B1.351 and Δ P.1. pools in volunteers after a single dose of BNT162b2 vaccine.** IFN-γ ELISpot responses to B. strain, B.1.1.7, B.1.351 and P.1. T cell responses in 5 healthy volunteers, 28 days after receiving a single dose of BNT162b2 vaccine. **a** T cell responses to 15-18-mer peptides in B. strain overlapping by 10 amino-acids and spanning the entire spike region **b** T cell responses to summed peptides from B. strain that mapped to sites with mutations in B.1.1.7 (n=17 peptides), B.1.351 (n=21 peptides) and P.1 (n=22 peptides). **c** Percentage contribution of SARS-CoV-2 variants B.1.1.7, B.1.351 and P.1 to the total T cell spike response in each of the 5 volunteers. Variants shown as a percentage of total spike response to B. strain as determined by ex vivo IFN-γ ELISpot SFC/$10^6$ PBMC = spot forming cells per million peripheral blood mononuclear cells, with DMSO background subtracted.

# Supplementary Figure 6

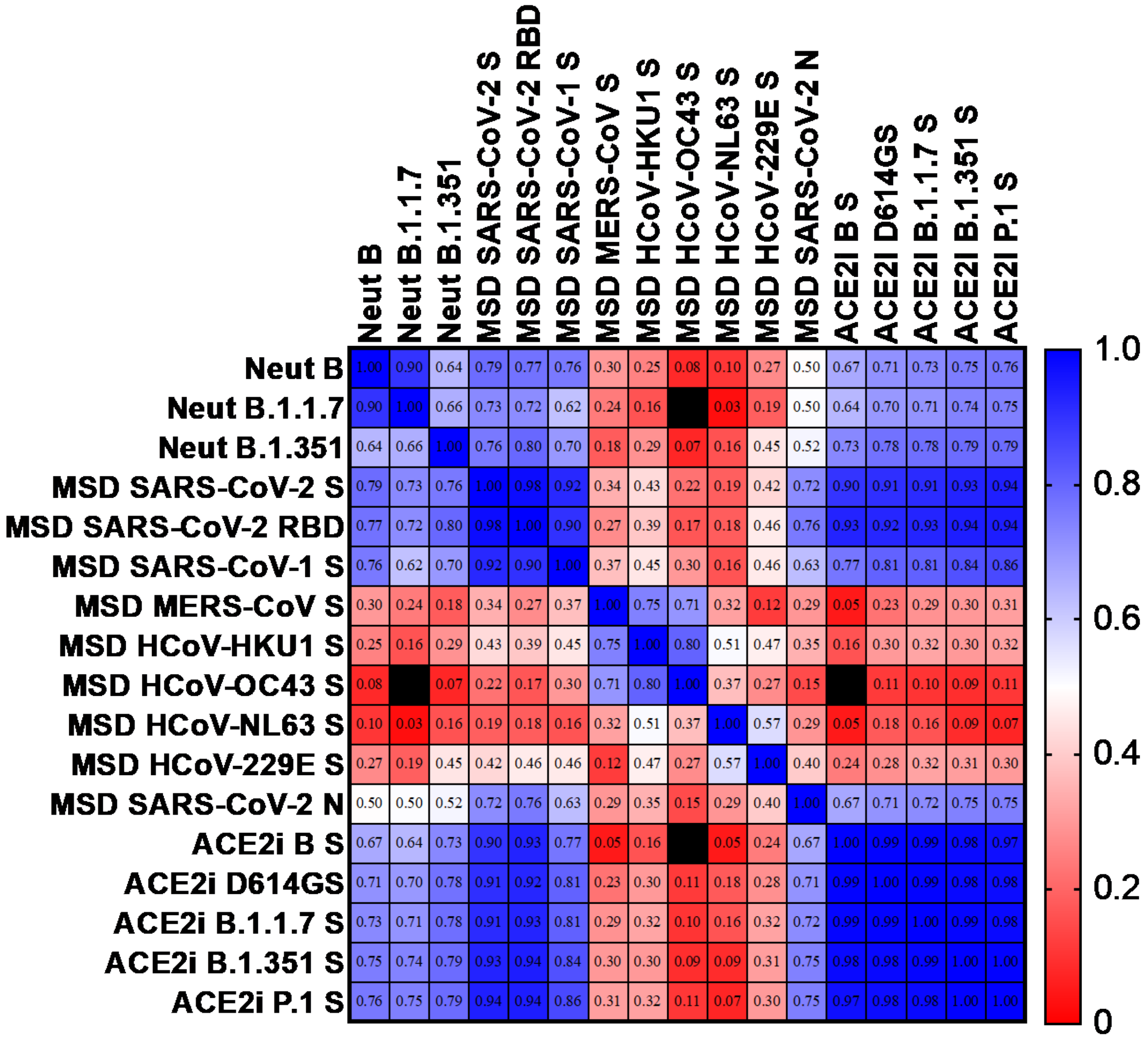


**Cross-correlation of immune parameters, including ELISpot, following two vaccine doses.**
For each serum and corresponding T cell sample, pairwise Spearman correlation analyses were undertaken between the value of binding of serum antibody to coronavirus antigens, the ACE2-spike binding-inhibition potency (see Figure 2), the homotypic and heterotypic neutralizing titre of the same sera (see Figure 3), and the degree of T cell responses to peptides in B strain spanning the entire spike region measured using IFN-γ ELISp assays (see Figure 4). Heatmap of Spearman's r parameter for each comparison in which spike binding data w available (n = 24) for individuals who received two doses of BNT162b2 vaccine (7-17 days after receiving the 2nd dose) . Colour mapping is dual gradient from Blue (r = 1.0) through White (r = 0.5) to Red (r = 0). Values outside this range are Black.

Supplementary Table 1: Individual vaccine and convalescent sample details.

## Vaccinees

**Pfizer samples post-prime**

| | *MSD Binding* | *MSD ACE2 Binding Inhibition* | *MNA* | *ELISpot* |
|---|---|---|---|---|
| n | 11 | 11 | 11 | 5 |
| mean | mean 29 days post-prime | nmean 29 days post-prime | mean 29 days post-prime | mean 35 days post-prime |
| range | 18-41 | 18-41 | 18-41 | 28-41 |

**Pfizer samples post-boost**

| | **Days between Prime & Boost** | *MSD Binding* | *MSD ACE2 Binding Inhibition* | *MNA* | *ELISpot* |
|---|---|---|---|---|---|
| n | 25 | 25 | 24 | 25 | 25 |
| mean | 24 | mean 8 days post-boost | mean 8 days post-boost | mean 8 days post-boost | mean 8 days post-boost |
| range | 18-28 | 07-17 | 07-12 | 07-17 | 07-17 |

## Convalescents

| | Severity | MSD Binding | MNA |
|---|---|---|---|
| n | mild | 62 | 13 |
| mean | | mean 29 days post symptom onset | mean 34 days post symptom onset |
| range | | 18-40 | 24-85 |
| | | | |
| n | Asymptomatic | 11 | 12 |
| mean | | mean 27 days post PCR Positivity | mean 30 days post PCR Positivity |
| range | | 22-33 | 22-61 |
| | | | |
| * 103 Pre-Pandemic samples run on MSD Binding Assays | | | |
| ** 4 infection naïve participants run on MNA | | | |
| *** 13 infection naïve participants run on ELISpots | | | |

Supplementary Table 1: Summary information for naïve vaccinated and convalescent individuals

Group numbers, mean and range (in terms of days) between BNT162b2vaccine doses and sample acquisition for assays performed for vaccinated individuals, and group number, mean and range (in terms of days) between symptom onset or PCR positivity and sample acquisition for assays performed for convalescent individuals.

| IDs | L18F | T20N | P26S | 69/70 | D80A | D138Y | Y144 | R190S | D215G | LH242 | R246I | K417N | E484K | N501Y | A570D | H655Y | P681H | A701V | T716I | S982A | T1027I | D1118H |
|---|---|---|---|---|---|---|---|---|---|---|---|---|---|---|---|---|---|---|---|---|---|---|
| 002 | 0 | 0 | 0 | 5 | 80 | 70 | 15 | 0 | 0 | 0 | 10 | 0 | 30 | 15 | 20 | 0 | 0 | 0 | 25 | 0 | 0 | 0 |
| 003 | 3 | 0 | 5 | 0 | 0 | 5 | 5 | 5 | 5 | 0 | 8 | 0 | 0 | 3 | 8 | 5 | 0 | 0 | 8 | 28 | 10 | 5 |
| 167 | 0 | 0 | 0 | 0 | 0 | 0 | 0 | 0 | 0 | 0 | 0 | 0 | 0 | 0 | 0 | 0 | 0 | 0 | 5 | 0 | 0 | 0 |
| 261 | 3 | 0 | 10 | 3 | 3 | 3 | 0 | 0 | 5 | 0 | 5 | 13 | 3 | 5 | 0 | 5 | 5 | 3 | 0 | 8 | 3 | 13 |
| 491 | 0 | 0 | 0 | 0 | 0 | 0 | 25 | 0 | 0 | 0 | 0 | 5 | 0 | 0 | 0 | 0 | 0 | 0 | 0 | 0 | 0 | 0 |
| 501 | 3 | 3 | 8 | 10 | 65 | 75 | 5 | 3 | 15 | 8 | 10 | 50 | 8 | 13 | 0 | 0 | 13 | 0 | 3 | 8 | 33 | 10 |
| 506 | 5 | 0 | 0 | 10 | 0 | 10 | 0 | 0 | 25 | 10 | 25 | 0 | 5 | 0 | 0 | 15 | 0 | 5 | 15 | 5 | 0 | 0 |
| 507 | 15 | 5 | 0 | 20 | 10 | 25 | 10 | 5 | 0 | 25 | 35 | 20 | 10 | 10 | 5 | 20 | 0 | 10 | 15 | 25 | 15 | 0 |
| 508 | 15 | 5 | 5 | 15 | 20 | 10 | 20 | 20 | 10 | 0 | 10 | 15 | 15 | 0 | 10 | 20 | 0 | 10 | 5 | 0 | 10 | 0 |
| 510 | 0 | 30 | 20 | 0 | 60 | 0 | 0 | 0 | 15 | 10 | 5 | 10 | 5 | 15 | 10 | 5 | 20 | 0 | 35 | 0 | 10 | 15 |
| 511 | 0 | 3 | 23 | 8 | 18 | 23 | 18 | 38 | 20 | 15 | 28 | 18 | 10 | 20 | 23 | 15 | 50 | 5 | 25 | 15 | 18 | 23 |
| 512 | 8 | 3 | 8 | 0 | 5 | 20 | 10 | 0 | 30 | 0 | 13 | 13 | 363 | 163 | 0 | 0 | 20 | 15 | 65 | 5 | 0 | 15 |
| 513 | 0 | 0 | 0 | 5 | 3 | 0 | 0 | 20 | 0 | 10 | 0 | 18 | 0 | 0 | 0 | 0 | 3 | 0 | 3 | 0 | 5 | 5 |
| 514 | 0 | 5 | 5 | 0 | 10 | 5 | 15 | 45 | 5 | 10 | 0 | 5 | 0 | 5 | 15 | 15 | 5 | 10 | 15 | 0 | 5 | 10 |
| 515 | 0 | 0 | 0 | 18 | 5 | 3 | 0 | 5 | 0 | 0 | 5 | 10 | 0 | 5 | 0 | 0 | 8 | 0 | 13 | 3 | 10 | 3 |
| 516 | 3 | 0 | 0 | 38 | 0 | 5 | 0 | 5 | 3 | 3 | 3 | 8 | 5 | 0 | 5 | 5 | 5 | 0 | 8 | 0 | 8 | 0 |
| 517 | 3 | 5 | 18 | 3 | 5 | 5 | 3 | 0 | 10 | 10 | 0 | 3 | 3 | 0 | 5 | 0 | 8 | 3 | 8 | 0 | 8 | 3 |
| 518 | 0 | 0 | 0 | 0 | 0 | 0 | 0 | 0 | 0 | 0 | 0 | 0 | 0 | 0 | 0 | 0 | 0 | 0 | 0 | 0 | 0 | 0 |
| 518 | 3 | 3 | 0 | 3 | 0 | 0 | 0 | 0 | 5 | 3 | 0 | 10 | 0 | 0 | 3 | 8 | 3 | 0 | 0 | 3 | 0 | 0 |
| 522 | 3 | 5 | 10 | 10 | 5 | 10 | 5 | 8 | 35 | 18 | 10 | 10 | 8 | 8 | 5 | 13 | 3 | 5 | 5 | 8 | 10 | 8 |
| 525 | 0 | 0 | 0 | 0 | 0 | 0 | 3 | 8 | 0 | 0 | 5 | 0 | 0 | 10 | 0 | 0 | 0 | 0 | 0 | 0 | 5 | 0 |
| 528 | 0 | 0 | 5 | 5 | 5 | 10 | 5 | 15 | 5 | 10 | 5 | 0 | 10 | 10 | 5 | 0 | 0 | 15 | 0 | 5 | 0 | 5 |
| 529 | 5 | 5 | 5 | 3 | 38 | 15 | 23 | 5 | 3 | 0 | 10 | 5 | 10 | 13 | 8 | 3 | 3 | 0 | 5 | 0 | 5 | 13 |
| 567 | 0 | 0 | 5 | 5 | 0 | 10 | 0 | 55 | 13 | 0 | 0 | 25 | 0 | 43 | 0 | 8 | 0 | 20 | 0 | 0 | 5 | 10 |

Supplementary Table 2: Individual SARS-CoV-2 vaccinee T cell epitope data.

Ex vivo IFN-γ ELISpot T cell responses to 22 mutation peptide sites relative to B strain are shown. Each row represents a single individual, and each column represents peptides in prototype strain that correspond with mutations in B.1.1.7, B.1.351 and P.1. in 24 volunteers, results reported as SFC/$10^6$ PBMC. SFC/$10^6$ PBMC = spot forming cells per million peripheral blood mononuclear cells, with DMSO background subtracted.

Tables S3: Cross-correlation of assays after one and two doses of vaccine and after SARS-CoV-2 infection: Spearman correlation parameters.

a

| *Correlation- Spearman r* | Neut B | Neut B.1.1.7 | Neut B.1.351 | MSD SARS-CoV-2 S | MSD SARS-CoV-2 RBD | MSD SARS-CoV-1 S | MSD MERS-CoV S | MSD HCoV-HKU1 S | MSD HCoV-OC43 S | MSD HCoV-NL63 S | MSD HCoV-229E S | MSD SARS-CoV-2 N |
|---|---|---|---|---|---|---|---|---|---|---|---|---|
| ***Neut B*** | 1.00 | 0.93 | 0.54 | 0.13 | 0.55 | 0.62 | 0.69 | 0.63 | 0.69 | 0.53 | 0.04 | 0.55 |
| ***Neut B.1.1.7*** | 0.93 | 1.00 | 0.58 | 0.27 | 0.56 | 0.56 | 0.72 | 0.59 | 0.68 | 0.56 | 0.12 | 0.55 |
| ***Neut B.1.351*** | 0.54 | 0.58 | 1.00 | 0.29 | 0.24 | 0.33 | 0.21 | 0.31 | 0.35 | 0.34 | 0.22 | 0.27 |
| ***MSD SARS-CoV-2 S*** | 0.13 | 0.27 | 0.29 | 1.00 | 0.39 | 0.40 | 0.23 | 0.40 | 0.23 | 0.23 | 0.44 | 0.34 |
| ***MSD SARS-CoV-2 RBD*** | 0.55 | 0.56 | 0.24 | 0.39 | 1.00 | 0.66 | 0.72 | 0.79 | 0.83 | 0.70 | 0.50 | 0.83 |
| ***MSD SARS-CoV-1 S*** | 0.62 | 0.56 | 0.33 | 0.40 | 0.66 | 1.00 | 0.78 | 0.96 | 0.71 | 0.43 | 0.38 | 0.78 |
| ***MSD MERS-CoV S*** | 0.69 | 0.72 | 0.21 | 0.23 | 0.72 | 0.78 | 1.00 | 0.82 | 0.78 | 0.36 | 0.27 | 0.71 |
| ***MSD HCoV-HKU1 S*** | 0.63 | 0.59 | 0.31 | 0.40 | 0.79 | 0.96 | 0.82 | 1.00 | 0.82 | 0.55 | 0.45 | 0.87 |
| ***MSD HCoV-OC43 S*** | 0.69 | 0.68 | 0.35 | 0.23 | 0.83 | 0.71 | 0.78 | 0.82 | 1.00 | 0.60 | 0.47 | 0.84 |
| ***MSD HCoV-NL63 S*** | 0.53 | 0.56 | 0.34 | 0.23 | 0.70 | 0.43 | 0.36 | 0.55 | 0.60 | 1.00 | 0.50 | 0.69 |
| ***MSD HCoV-229E S*** | 0.04 | 0.12 | 0.22 | 0.44 | 0.50 | 0.38 | 0.27 | 0.45 | 0.47 | 0.50 | 1.00 | 0.55 |
| ***MSD SARS-CoV-2 N*** | 0.55 | 0.55 | 0.27 | 0.34 | 0.83 | 0.78 | 0.71 | 0.87 | 0.84 | 0.69 | 0.55 | 1.00 |

b

| *Correlation- Spearman r* | *Neut B* | *Neut B.1.1.7* | *Neut B.1.351* | MSD SARS-CoV-2 S | MSD SARS-CoV-2 RBD | MSD SARS-CoV-1 S | MSD MERS-CoV S | MSD HCoV-HKU1 S | MSD HCoV-OC43 S | MSD HCoV-NL63 S | MSD HCoV-229E S | MSD SARS-CoV-2 N | ACE2i B S | ACE2i D614GS | ACE2i B.1.1.7 S | ACE2i B.1.351 S | ACE2i P.1 S |
|---|---|---|---|---|---|---|---|---|---|---|---|---|---|---|---|---|---|
| *Neut B* | 1.00 | 0.75 | -0.14 | 0.39 | 0.35 | 0.34 | -0.32 | 0.07 | -0.04 | -0.01 | 0.48 | -0.26 | 0.19 | 0.15 | 0.09 | 0.18 | 0.18 |
| *Neut B.1.1.7* | 0.75 | 1.00 | 0.07 | 0.21 | 0.26 | 0.10 | -0.27 | 0.00 | -0.16 | 0.00 | 0.40 | 0.02 | 0.08 | 0.14 | 0.10 | 0.13 | 0.13 |
| *Neut B.1.351* | -0.14 | 0.07 | 1.00 | 0.41 | 0.50 | 0.18 | -0.14 | 0.30 | 0.16 | 0.29 | 0.44 | 0.26 | 0.38 | 0.45 | 0.41 | 0.26 | 0.26 |
| *MSD SARS-CoV-2 S* | 0.39 | 0.21 | 0.41 | 1.00 | 0.95 | 0.93 | 0.15 | 0.68 | 0.68 | 0.13 | 0.78 | 0.30 | 0.65 | 0.57 | 0.53 | 0.62 | 0.62 |
| *MSD SARS-CoV-2 RBD* | 0.35 | 0.26 | 0.50 | 0.95 | 1.00 | 0.80 | -0.04 | 0.55 | 0.51 | 0.16 | 0.82 | 0.38 | 0.59 | 0.53 | 0.51 | 0.55 | 0.55 |
| *MSD SARS-CoV-1 S* | 0.34 | 0.10 | 0.18 | 0.93 | 0.80 | 1.00 | 0.45 | 0.82 | 0.85 | 0.15 | 0.72 | 0.35 | 0.50 | 0.46 | 0.40 | 0.53 | 0.53 |
| *MSD MERS-CoV S* | -0.32 | -0.27 | -0.14 | 0.15 | -0.04 | 0.45 | 1.00 | 0.65 | 0.73 | -0.08 | -0.04 | 0.39 | -0.04 | 0.07 | 0.05 | 0.11 | 0.11 |
| *MSD HCoV-HKU1 S* | 0.07 | 0.00 | 0.30 | 0.68 | 0.55 | 0.82 | 0.65 | 1.00 | 0.84 | 0.35 | 0.55 | 0.35 | 0.35 | 0.45 | 0.37 | 0.35 | 0.35 |
| *MSD HCoV-OC43 S* | -0.04 | -0.16 | 0.16 | 0.68 | 0.51 | 0.85 | 0.73 | 0.84 | 1.00 | 0.06 | 0.51 | 0.29 | 0.17 | 0.16 | 0.11 | 0.21 | 0.21 |
| *MSD HCoV-NL63 S* | -0.01 | 0.00 | 0.29 | 0.13 | 0.16 | 0.15 | -0.08 | 0.35 | 0.06 | 1.00 | 0.44 | 0.37 | 0.01 | 0.15 | 0.03 | -0.04 | -0.04 |
| *MSD HCoV-229E S* | 0.48 | 0.40 | 0.44 | 0.78 | 0.82 | 0.72 | -0.04 | 0.55 | 0.51 | 0.44 | 1.00 | 0.30 | 0.16 | 0.16 | 0.07 | 0.14 | 0.14 |
| *MSD SARS-CoV-2 N* | -0.26 | 0.02 | 0.26 | 0.30 | 0.38 | 0.35 | 0.39 | 0.35 | 0.29 | 0.37 | 0.30 | 1.00 | 0.25 | 0.36 | 0.37 | 0.41 | 0.41 |
| *ACE2i B S* | 0.19 | 0.08 | 0.38 | 0.65 | 0.59 | 0.50 | -0.04 | 0.35 | 0.17 | 0.01 | 0.16 | 0.25 | 1.00 | 0.95 | 0.95 | 0.96 | 0.96 |
| *ACE2i D614GS* | 0.15 | 0.14 | 0.45 | 0.57 | 0.53 | 0.46 | 0.07 | 0.45 | 0.16 | 0.15 | 0.16 | 0.36 | 0.95 | 1.00 | 0.98 | 0.94 | 0.94 |
| *ACE2i B.1.1.7 S* | 0.09 | 0.10 | 0.41 | 0.53 | 0.51 | 0.40 | 0.05 | 0.37 | 0.11 | 0.03 | 0.07 | 0.37 | 0.95 | 0.98 | 1.00 | 0.95 | 0.95 |
| *ACE2i B.1.351 S* | 0.18 | 0.13 | 0.26 | 0.62 | 0.55 | 0.53 | 0.11 | 0.35 | 0.21 | -0.04 | 0.14 | 0.41 | 0.96 | 0.94 | 0.95 | 1.00 | 1.00 |
| *ACE2i P.1 S* | 0.18 | 0.13 | 0.26 | 0.62 | 0.55 | 0.53 | 0.11 | 0.35 | 0.21 | -0.04 | 0.14 | 0.41 | 0.96 | 0.94 | 0.95 | 1.00 | 1.00 |

**Supplementary Table 3: Cross-correlation of assays after one dose of vaccine and after SARS-CoV-2 infection: Spearman correlation parameters**.

For each serum, pairwise Spearman correlation analyses were undertaken between the value of binding of serum antibody to coronavirus antigens, the ACE2-spike binding-inhibition potency (see Figure 2), and the homotypic and heterotypic neutralizing titre of the same sera (see Figure 3). **a** Table displaying two-tailed Spearman's r parameter for each comparison for samples taken after SARS-CoV-2 infection (n = 20, 11:9 asymptomatic to symptomatic, mean 28 days since PCR test for asymptomatics or symptoms onset) with the exception of ACE2-spike binding-inhibition**. b** Table displaying two-tailed Spearman's r parameter for each comparison for samples taken after one vaccine dose (n = 11, mean 27 days post vaccination).
