# Supplementary material for: Two doses of SARS-CoV-2 vaccination induce robust immune responses to emerging SARS-CoV-2 variants of concern
Source: Nat Commun. 2021 Aug 17;12:5061. doi: 10.1038/s41467-021-25167-5 (PMC8371089; doi:10.1038/s41467-021-25167-5)
Supplement: Supplementary file 3 — Reporting summary [file 41467_2021_25167_MOESM3_ESM.pdf]

nature portfolio

Corresponding author(s): William James, Miles Carroll and Eleanor Barnes

Last updated by author(s): Jun 19, 2021

Reporting Summary

Nature Portfolio wishes to improve the reproducibility of the work that we publish. This form provides structure for consistency and transparency in reporting. For further information on Nature Portfolio policies, see our Editorial Policies and the Editorial Policy Checklist.

Statistics

For all statistical analyses, confirm that the following items are present in the figure legend, table legend, main text, or Methods section.

n/a	Confirmed	
☐	☒	The exact sample size (n) for each experimental group/condition, given as a discrete number and unit of measurement
☐	☒	A statement on whether measurements were taken from distinct samples or whether the same sample was measured repeatedly
☐	☒	The statistical test(s) used AND whether they are one- or two-sided<br>*Only common tests should be described solely by name; describe more complex techniques in the Methods section.*
☐	☒	A description of all covariates tested
☐	☒	A description of any assumptions or corrections, such as tests of normality and adjustment for multiple comparisons
☐	☒	A full description of the statistical parameters including central tendency (e.g. means) or other basic estimates (e.g. regression coefficient) AND variation (e.g. standard deviation) or associated estimates of uncertainty (e.g. confidence intervals)
☐	☒	For null hypothesis testing, the test statistic (e.g. F, t, r) with confidence intervals, effect sizes, degrees of freedom and P value noted<br>*Give P values as exact values whenever suitable.*
☒	☐	For Bayesian analysis, information on the choice of priors and Markov chain Monte Carlo settings
☒	☐	For hierarchical and complex designs, identification of the appropriate level for tests and full reporting of outcomes
☐	☒	Estimates of effect sizes (e.g. Cohen's d, Pearson's r), indicating how they were calculated

Our web collection on statistics for biologists contains articles on many of the points above.

Software and code

Policy information about availability of computer code

Data collection	ELISpot - AID ELISpot Reader (v4.0)
Data analysis	Data were analysed using GraphPad Prism V9.01

For manuscripts utilizing custom algorithms or software that are central to the research but not yet described in published literature, software must be made available to editors and reviewers. We strongly encourage code deposition in a community repository (e.g. GitHub). See the Nature Portfolio guidelines for submitting code & software for further information.

Data

Policy information about availability of data

All manuscripts must include a data availability statement. This statement should provide the following information, where applicable:

- Accession codes, unique identifiers, or web links for publicly available datasets
- A description of any restrictions on data availability
- For clinical datasets or third party data, please ensure that the statement adheres to our policy

The data generated in this study are provided in the Supplementary Information/Source Data file.

Field-specific reporting

Life sciences study design

All studies must disclose on these points even when the disclosure is negative.

Sample size	No sample size calculations were performed. This study developed and was developed ad hoc as the UK vaccination programme for healthcare workers was commenced in our University hospitals. Concomitantly, the emerging variants of concern were recognised. Accordingly we recruited any consenting HCWs where resources allowed. We recruited HCW who had recieved one and two doses (n=11 and 25 respectively, 11 overlapped the groups). We aimed to study the impact of VOC on antibody and T cell responses after vaccination and wanted to compare it to those with natural infection. HCWs with previous infection had been recruited as part of a cohort study. 77 of these samples (12 asymptomatic and 65 mild infection samples) were used. Again these numbers were not based on power calucation, rather sample availability. 13 unvaccinated participants were used as controls for T cell studies, 4 for neutralizing antibody study controls and 103 pre-pandemic samples used as controls for the MSD assays. The control sample numbers were not based on power calculations but resource availability, and assay and time constraints. The purpose was to generate immunological insights as resources allowed. These sample numbers are not uncommon for human adaptive immune studies
Data exclusions	All vaccinated subjects who were recruited were included. 1 vaccinated HCWs t cell results was excluded due to failure of quality controls. All controls run on assays were used in results.
Replication	Standardised ELISpot assays were run in triplicate for background and spike peptides and duplicates for all others to allow cell preservation. DMSO control with matching percent DMSO was also used in all assays to account for DMSO content in peptide pools. MSD ACE2 inhibition assay was run in monoplicate. MSD serology assay was run in monoplicate (convalescent samples) or duplicates (vaccinee samples). MNA tests were performed in quadruplicate for all samples. Each plate contained serum-free controls for normalization of results. Where multiplicates were run, all were successful.
Randomization	We performed no randomisation. Samples were selected based on vaccine history and convalescence.
Blinding	Blinding was not possible or relevant for this study looking at the impact of VOC on antibody and T cell responses to spike protein.

Reporting for specific materials, systems and methods

We require information from authors about some types of materials, experimental systems and methods used in many studies. Here, indicate whether each material, system or method listed is relevant to your study. If you are not sure if a list item applies to your research, read the appropriate section before selecting a response.

Materials & experimental systems

n/a	Involved in the study	
☐	☒	Antibodies
☒	☐	Eukaryotic cell lines
☒	☐	Palaeontology and archaeology
☒	☐	Animals and other organisms
☐	☒	Human research participants
☒	☐	Clinical data
☒	☐	Dual use research of concern

Methods

n/a	Involved in the study	
☒	☐	ChIP-seq
☒	☐	Flow cytometry
☒	☐	MRI-based neuroimaging

Antibodies

Antibodies used	anti-IgG SULFO-TAG (cat. no: R32AJ-1: Meso Scale Diagnostics, Rockville, MD USA) for the MSD serology assay, Used as part of the MSD V-PLEX COVID-19 Coronavirus Panel 3 (IgG) Kit (cat. no. K15399U). GZ-4 antihuman IFN-γ (Mabtech, AB, Sweden) was used to coat ELISpot plate, anti-IFN-γ biotinylated mAb (7-B6-1-biotin, Mabtech) as secondary antibody. FI 3A, GR 12C, FD 11A, EY-2A and EY-6A were isolated in Alain Townsend's lab and steps are described in Huang, et al. (2020). Plasmablast-derived antibody response to acute SARS-CoV-2. Plos Pathogen (2021). C121 was derived by gene synthesis (GeneArt) from the published sequence in Robbiani et al. Convergent antibody responses to SARS-CoV-2 in convalescent individuals. Nature 584, 437–442 (2020) S309 was derived by gene synthesis (GeneArt) from the published sequence in Pinto, D. et al. Cross-neutralization of SARS-CoV-2 by a human monoclonal SARS-CoV antibody. Nature 583, 290–295 (2020). Goat Anti-Human IgG (Fc-specific)–Peroxidase Sigm-Aldrich, Germany (cat. no. A0170-1ML). National Institute for Biological Standards and Control (NIBSC) 20/130 reference plasma was obtained from the NIBSC, UK. It is human plasma from a donor recovered from COVID-19.Flow Cytometry antibodies used to develop monoclonal antibodies – as previously reported and not specifically performed again for this study: Pacific Blue anti-CD3 (clone UCHT1, cat. no. 558117, 420 BD), fluorescein isothiocyanate anti-CD19 (clone HIB19, cat. no. 555412, BD), 421 phycoerythrin-Cy7 anti-CD27 (clone M-T271, cat. no. 560609, BD), 422 allophycocyanin-H7 anti-CD20 (clone L27, cat. no. 641396, BD), phycoerythrin423 Cy5 anti-CD38 (clone HIT2, cat. no. 555461, BD) and phycoerythrin anti-human IgG (clone G18-145, cat. no. 555787, BD).
Validation	All antibodies used were tested with appropriate negative and positive control sample. Links below to manufacturer pages with statements where given and/or original papers where antibodies used are first described.<br>Manufacturer pages with statements and or original papers where antibodies first described:<br>https://www.mesoscale.com/products/anti-human-antibody-goat-sulfo-tag-labeled-r32aj/<br>https://www.mesoscale.com/en/products/v-plex-covid-19-coronavirus-panel-3-igg-kit-5-pl-k15399u/

https://www.thermofisher.com/antibody/product/IFN-gamma-Antibody-clone-GZ-4-Monoclonal/BMS107
https://www.mabtech.com/products/anti-human-ifn-gamma-antibody-7-b6-1-biotinylated-3420-6#tabs-min-2
https://www.sigmaaldrich.com/GB/en/product/sigma/a0170# anti-hu IgG
https://www.nature.com/articles/s41586-020-2456-9#Sec22 : C121
https://www.nature.com/articles/s41586-020-2349-y : S309
https://doi.org/10.1371/journal.ppat.1009352 : FI 3A, GR 12C, FD 11A, EY-2A and EY-6A
https://www.nibsc.org/products/brm_product_catalogue/detail_page.aspx?catid=20/130 - NIBSC/ WHO standard
https://www.nature.com/articles/s41564-018-0303-7 flow cytometry antibody descriptions and gating strategies which were not rerun specifically for this study.

Human research participants

Policy information about studies involving human research participants

Population characteristics	Healthcare workers between the ages of 26 and 68 who had mild or asymptomatic SARS-CoV-2 infection or were infection naive and recieved BNT162b2 Pfizer/BioNTech vaccination. 66% female and 33% male. Convalescents who experienced severe disease were between the ages of 37 and 53 years and F:M 6:2
Recruitment	HCWs who expressed interested in being contacted for SARS-CoV-2 research were recruited in our University hospitals after a PCR positive test result or before or after vaccination with BNT162b2 mRNA vaccine. Alternative controls were recruited in a similar manner in Sheffield. Pre-pandemic samples were from a previous vaccine study, performed in London in 2017. With regards the infection or vaccine participants, those who read emails/ engage with staff messaging were more likely to take part. This may have lead to self-selection bias in that the recruited participants may be more homogeneous than if randomly selected from staff. We do not believe that these factors would have greatly affected immunological responses to either infection or vaccination. Immune response to vaccination seems to tally with published RCT data and previous infection responses are in keeping with other published studies.
Ethics oversight	Healthcare Workers (HCWs) with mild and asymptomatic SARS-CoV-2 infection and infection naïve HCWs, either before or after vaccination with BNT162b2, were recruited under the OPTIC Study: Oxford Translational Gastrointestinal Unit GI Biobank Study 16/YH/0247 [REC at Yorkshire & The Humber – Sheffield]. Four additional unvaccinated participants were recruited under the Observational Biobanking study approvals SthObs (18/YH/0441). Pre-pandemic negative control sera were obtained from a prior vaccine study of the National Vaccine Evaluation Consortium, performed in 2017 under ethical approval from NHS Heath Research Authority – NRES committee London City and East 2017.

Note that full information on the approval of the study protocol must also be provided in the manuscript.
